# Benefits of an online group dance program for adolescents' social bonding and wellbeing

**DOI:** 10.1002/jad.12391

**Published:** 2024-08-15

**Authors:** Bahar Tunçgenç, Emma‐Jane Greig, Emma Cohen

**Affiliations:** ^1^ Psychology Department Nottingham Trent University Nottingham UK; ^2^ Institute of Human Sciences University of Oxford Oxford UK; ^3^ Body Politic Dance Limited Oxford UK

**Keywords:** adolescence, dance, mental health, social bonding, social connectedness, wellbeing

## Abstract

**Introduction:**

The Covid‐19 pandemic and its ensuing effects on mental health made it clearer than ever that social bonds are critical for survival, flourishing, and mental wellbeing. Experimental laboratory‐based research with children and adults shows that activities involving movement alignment and joint action, such as dance, can facilitate social bonds.

**Methods:**

This study examined whether an online group dance intervention positively affects social bonding and wellbeing using a randomized control design. Participants were 58 UK adolescents aged 11–16 years (*N* = 52 girls, 75% White, 7% Asian/Asian British, 18% Mixed‐Other), randomly assigned to an online intervention or waitlist control group. Participants in the intervention group completed an online 5‐week hip‐hop dance program during the Covid‐19 pandemic in January to February 2021. Measures of social bonding, wellbeing, and future orientation were taken at the beginning and end of the program.

**Results:**

Linear mixed model analyses examining group × timepoint interaction showed greater increase in social bonding (*p* < .0001), and wellbeing (*p* < .0001) in the intervention vs control group. Moreover, increases in bonding significantly predicted increases in wellbeing (*p* < .0001), and increases in bonding (*p* = .03) and wellbeing (*p* = .0002) significantly predicted increases in the adolescents' hope for the future.

**Conclusions:**

These data, collected at a time of mass social isolation, show that a 5‐week‐long online dance activity can help adolescents forge stronger social bonds, and improve their wellbeing and future orientation. Our findings suggest that the wellbeing and future orientation benefits of group dance may stem from having stronger social connectedness, opening up avenues for future research and interventions.

## 1 INTRODUCTION

**Figure 1:**
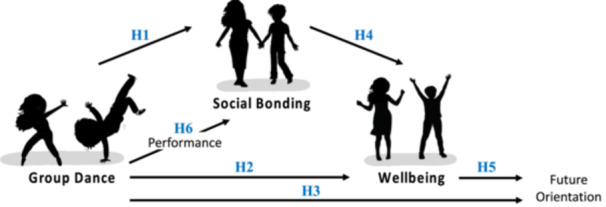
Theoretical model and hypotheses (H1–H6) tested in this study. The model proposes that group dance promotes social bonding, which, in turn, improves wellbeing. In addition, subjective perceptions of performance will positively predict social bonding, and improved wellbeing will positively predict future orientation.

Group dance is observed in all known human cultures to date (Ehrenreich, 2006), with precursors of dance emerging early in development as babies sway, tap, or rock their bodies to align their movements with external rhythms (Cirelli, 2018). Dancing and musicality, more broadly, are proposed to have deep evolutionary roots in human social behavior through their bonding function within groups (Savage et al., 2021). If group dance is such an evolutionarily and cross‐culturally pervasive activity, what benefits could it have for contemporary issues around social connectedness and mental wellbeing? According to national data (NHS England, 2018, 2021), one in six young people in the United Kingdom experience a mental health problem, with most mental illnesses being established by age 14. Moreover, people with a mental illness are twice as likely to feel lonely most or all the time. Thus, it is pertinent to examine how group dance can help foster social connectedness and sustain mental wellbeing. Here, using a randomized control experimental design, we assess a theoretical model (see Figure 1) that asserts that: (i) engaging in group dance will promote social bonding (Savage et al., 2021) and (ii) social bonding induced by group dance will lead to improved wellbeing and future orientation (Fancourt et al., 2021).

### 1.1 Social bonding effects of dance

A defining feature of group dance is tight movement alignment between dancers and the external rhythms. A rich body of experimental research with children and adults shows that such tight alignment in the form of rhythmic synchronization facilitates prosociality, including enhanced feelings of closeness, interpersonal liking, trust, and cooperative behaviors such as sharing with or helping others (Mogan et al., 2017; Rennung & Göritz, 2016; Tarr et al., 2015). Beyond dyadic interactions, dancing has been shown to induce social bonding effects at group level (Jackson et al., 2018; Mogan et al., 2017). For instance, groups of strangers asked to dance synchronously with each other reported feeling as a single unit with their fellow dancers (Reddish et al., 2013; Tarr et al., 2016). In children, it has been shown that children who were initially split into opposing groups no longer held negative feelings or attitudes towards the other group's members upon dancing in synchrony with them (Tunçgenç & Cohen, 2016). Yet, we do not know how well these findings obtained in relatively confined laboratory settings apply to real‐life situations or what their utility is in addressing pressing mental wellbeing issues.

Group dance is proposed to have these facilitatory bonding effects as it taps into biological, psychological, and social processes that promote social connectedness (Fancourt et al., 2021). At the neurobiological and psychological level, dancing requires multisensory processing to combine the auditory cues of the rhythms with the visual cues of others' movements and the proprioceptive cues of one's own movements (Kaeppler, 1978). By dancing to the same rhythm, people experience sensory‐motor coupling, which can lead to a perceived overlap between the self and the other, and feelings of increased closeness (Smith, 2008; Tunçgenç, 2023). Dancing also activates the endocannabinoid and opioid systems, leading participants to feel less pain (Tarr et al., 2015, 2016), and is often associated with enjoyment, relaxation, and happiness (Mansfield et al., 2018). In addition, performance factors, such as moderate levels of exertion (Davis et al., 2015; Tarr et al., 2015) and subjective ratings of group performance (Cohen et al., 2023) in dance and physical activity have been linked to positive effects on social bonding and cooperation, potentially via activation of the endocannabinoid and opioid systems. This experimental developmental and social psychology literature motivates the first part of our theoretical model (Figure 1) that group dancing promotes social bonding.

### 1.2 Wellbeing effects of social bonding and dance

A large epidemiological literature shows that social factors critically influence people's health behavior and physical and mental health outcomes. Broadly construed as the “social determinants of health,” these conditions include “proximate” daily life circumstances, such as family or community relationships, as well as “distal” societal structures, such as education, economic, or social welfare systems (Solar & Irwin, 2010). It has been shown that having strong social bonds with one's family, friends, and community positively impacts wellbeing through promoting healthy behavior, access to facilities, and emotional support, as well as through shaping group identities within communities (Jetten et al., 2014).

A key social determinant of health for adolescents is peer relationships (Irwin et al., 2007; Viner et al., 2012), which are pivotal in young people's learning about social norms and identity formation. For instance, it has been shown that school belonging and strong peer relationships predict fewer mental health problems and better wellbeing in adolescents (Arslan, 2021). During the Covid‐19 pandemic, peer relationships impacted how much adolescents engaged in health behaviors and received social support, which, in turn, influenced their physical and mental health (Andrews et al., 2020). Hence, it is possible that activities such as group dance, which improves social bonding with peers, can have a positive impact on wellbeing and future orientation.

Supporting this premise, a recent review has shown that nonclinical arts‐based interventions have medium to strong effects on increasing subjective wellbeing (Blodgett et al., 2022). Despite being scarce and of variable quality (e.g., due to lacking control groups or having a small sample size), studies examining dance interventions revealed the benefits of group dance on improving depressive symptoms, wellbeing, social connectedness, and happiness (Mansfield et al., 2018; McCrary et al., 2021). In addition, participating in dance activities can positively impact children and young people's confidence, self‐esteem, social relationships, and belonging (Tao et al., 2022; Zarobe & Bungay, 2017). Overall, this social, epidemiological, and arts intervention literature motivates the second part of our theoretical model (Figure 1) that social bonding induced by group dance improves positive wellbeing and future orientation.

### 1.3 Current study

Despite existing studies showing promising results, the causal pathways linking dance and wellbeing are largely unknown (McCrary et al., 2021). To establish the effectiveness of group dance interventions for wellbeing, we need longitudinal, randomized controlled studies assessing causal mechanisms. Another important gap in our knowledge is on the utility of online programs for improving wellbeing. Since the Covid‐19 pandemic, there has been increased engagement with arts and clinical treatments through the online medium, especially by people with disabilities and mental illnesses (Bu et al., 2022). Given a range of personal and systemic factors that can limit public participation in arts (Ateca‐Amestoy & Castiglione, 2023), online programs have been proposed as a potential avenue for improving accessibility. It has been suggested that online arts and wellbeing interventions can be especially useful for young people, as they tend to have high digital literacy (Moss et al., 2023). Although some qualitative data suggest that online arts engagement can elicit feelings of togetherness and social connection (Perkins et al., 2022), our knowledge of the effectiveness of online dance programs is highly limited.

To begin to fill these gaps, this study examined the impact of a group dance program on adolescents' social bonding, wellbeing, and future orientation using a longitudinal, randomized controlled design. Participants were randomly assigned either to a 5‐week dance intervention group or a waitlist control group. Measures were taken at the beginning (Timepoint 1: T1) and at the end of the program (Timepoint 2: T2). We hypothesized that after taking part in the dance program, participants in the intervention group would display a greater increase in (i) social bonding with their peers, (ii) wellbeing, and (iii) future orientation as compared to participants in the waitlist control group. In addition, we hypothesized that for the dance program participants, (iv) social bonding and positive experiences during the program would positively predict increases in wellbeing, (v) wellbeing and social bonding would positively predict increases in future orientation, and (vi) group performance would positively predict increases in social bonding. All hypotheses, methods, and analyses of this study were preregistered before data collection: https://osf.io/b5dfp/.

## 2 MATERIALS AND METHODS

### 2.1 Participants

Participants were 58 adolescents, aged 11–16 years old, randomly assigned to the online dance intervention or waitlist control group. The group assignment was done by matching participant names with a number and drawing the numbers using a free online random number generator. Participants were mostly female, had a White ethnic background, and were residents in different parts of the United Kingdom, from Dundee (furthest north) to Portsmouth (furthest south) and Swansea (furthest west) to Colchester (furthest east). See Table 1 for detailed sample characteristics of each group.

**Table 1:** Descriptive statistics (*N* for group sample size, gender, and ethnicity; *M*, SD for all other variables) of sample characteristics and key variables from the original data set.

	T1			T2	
	Intervention	Waitlist		Intervention	Waitlist
Group sample size	31	27		13	20
Gender (girls/boys)	30/1	22/5		12/1	15/5
Ethnicity (White/non‐White)^a^	22/9	22/5		8/5	16/4
Age	13.67 (1.54)	13.11 (1.68)		13.98 (2.04)	13.30 (1.85)
Social bonding	23.95 (21.13)	24.33 (18.39)		52.22 (23.44)	16.60 (14.55)
Wellbeing	67.29 (16.75)	71.23 (15.63)		79.46 (10.53)	63.35 (20.70)
Future outlook	68.03 (26.40)	63.09 (23.38)		71.92 (21.75)	61.43 (22.70)
Hope	68.25 (24.55)	74.67 (22.60)		88.14 (17.49)	84.15 (16.67)
Positive experience	2.81 (0.43)	2.92 (0.35)		2.99 (0.55)	3.18 (0.35)
Performance expectations: Group	–	–		67.78 (20.22)	–
Performance satisfaction: Group	–	–		75.61 (28.83)	–
Performance expectations: Own	–	–		62.56 (19.40)	–
Performance satisfaction: Own	–	–		72.78 (23.92)	–

^a^ Non‐White ethnicity category included the sub‐categories of (a) Asian, Asian British, (b) Black, African, Caribbean, Black British, (c) Mixed, and (d) Other. These categories were taken from the Census for England conducted by the Office for National Statistics.

Participants were recruited on a voluntary basis from the existing pool of young people who had signed up to join the free dance program delivered by the Body Politic Dance Ltd. in January to February 2021. A total of 64% of participants (*N* = 37) attended at least four of the five weekly sessions, with 34% (*N* = 20) not missing any sessions. From conception onwards, this study was coproduced with the Body Politic Dance Ltd. at the partnership level of Arnstein's ladder of participation (Arnstein, 1969), with the owner of the company and the lead dancer being a coauthor in this paper (E‐J. G.).

There was a 43% dropout rate from T1 (*N* = 58) to T2 (*N* = 33) due to some participants not being present in the last session when T2 data was collected. Given the high dropout rate from T1 to T2, we used multiple imputation to be sufficiently powered in our inferential analyses (see details in Section 2.3). Thus, the final sample size used in inferential statistics was *N* = 58. Table 1 shows full descriptive statistics of key variables from the original data set, and Supporting Information S1: Table S1 shows the same descriptive statistics for the 10 imputed data sets.

This study received ethical approval from the School of Anthropology and Museum Ethnography Research Ethics Committee of the University of Oxford (Reference No: SAME_C1A_20_099). Written consent was obtained from all participants' parents/guardians, and verbal assent was received from the participants before data collection. Participants received the dance program without any interruptions even if they did not take part in the research study. Participants in the waitlist control group received the dance program at the end of the 5‐week period.

### 2.2 Measures and procedure

Details of all survey items are given below. To assess the internal reliability of all scales, we calculated omega values. For scales that were not standardized (i.e., social bonding and positive experience scales), we also conducted a confirmatory factor analysis (CFA). Following recommendations from prior literature (Hu & Bentler, 1999; Xia & Yang, 2019), a model was considered to be a good fit if it had a nonsignificant *χ* ^2^ result for the user model test, root mean square error of approximation (RMSEA) and standardized root mean square residual (SRMR) values less than 0.05–0.08, and comparative fit index (CFI) and Tucker–Lewis index (TLI) values greater than 0.90–0.95.

#### 2.2.1 Dance intervention

The dance program was delivered first to the intervention group, and then to the waitlist control group. Participants assigned to the waitlist control group were told that due to capacity reasons, their program was going to commence in 5 weeks' time and that, in the meantime, we would ask them to fill out some surveys for the research study. There were no further instructions or constraints imposed around waitlist participants' daily activities while they waited for their program to commence.

The dance program was delivered by three teachers from Body Politic Dance Ltd. Participants were emailed a Zoom link to attend the weekly 1‐hour‐long sessions. The sessions were split into check‐in, warm‐up, learning choreography, a creative task, and good‐bye phases. To mimic a similar structure to in‐person delivery and encourage participants to talk and feel connected with one another, the participants were encouraged to keep their cameras on, and breakout rooms were utilized (see more details below). The teachers constantly monitored the participants and adjusted the session as needed, for example, by slowing movements down or re‐explaining. Praise, positive feedback, and encouragement were given throughout sessions, though these were mostly directed to the group as a whole rather than to individuals due to the online format.

The movements in the choreography were rooted in exploring hip‐hop dance foundations and included both standing and floor work. The moves were linked together to form short dynamic pieces of choreography that would translate well over the screen. For the creative task, participants picked an image from a bank of interesting images found by the teachers. The images were an assortment of shapes, textures, and styles that were suggestive of mental health pressures and difficulties and that inspired movement. While the teachers did not explicitly discuss mental health with participants, the moves and themes helped young people express their own thoughts and feelings on this topic. After selecting their image, the participants were asked to create four “still poses” inspired by the physical shape of the image they selected or a feeling they experienced as they viewed the image. Then, participants shared and taught their poses to other participants in breakout rooms. These smaller breakout groups proved to be an effective way of getting young people to talk and interact with one another.

#### 2.2.2 Social bonding

Four items used with children and adolescents in previous research (Cohen et al., 2023) examined (i) how connected, (ii) how bonded, (iii) how committed, and (iv) how much like friends the adolescents felt towards the other participants at two timepoints, i.e., before and after the program. Each item was rated on a continuous scale ranging from 0 = *Not at all* through 50 = *As expected* to 100 = *Very much*. CFA confirmed that all four items loaded onto a single factor, with the *χ* ^2^ test being nonsignificant (*χ* ^2^[2] = 1.17, *p* = .56), RMSEA = 0.00, SRMR = 0.01, CFI = 1.00, and TLI = 1.01. Furthermore, the scale had excellent internal reliability (*ω* = 0.93, SE = 0.02, 95% confidence interval [CI]: 0.90–0.96). Thus, the mean of all four items was calculated to obtain the social bonding score, with higher scores indicating more bonding.

#### 2.2.3 Wellbeing

The 15‐item brief PERMA Profiler (Butler & Kern, 2016), addressing five components of wellbeing, that is, positive emotion, engagement, relationships, meaning, and accomplishment, was administered at both timepoints. Each item was rated on a continuous scale ranging from 0 = *Not at all/Never* to 100 = *Completely/Always*. The scale has been demonstrated to have good internal validity (Cronbach's *α* = .71–.94), test–retest reliability (Pearson *r* = 0.62–0.88), and model fit (*χ* ^2^[80] = 10.61, RMSEA = 0.06, SRMR = 0.03, CFI = 0.97, TLI = 0.96) in multinational samples of over 38,000 people (Butler & Kern, 2016). This measure has been successfully used with adolescents aged 12–19 in previous research (Kern et al., 2015; Singh & Raina, 2020; Waigel & Lemos, 2023). In our sample, the scale had excellent internal reliability (*ω* = 0.94, SE = 0.01, 95% CI: 0.92–0.97). Following scale guidelines, an overall wellbeing score was calculated by taking the mean of all items, with higher scores indicating more positive wellbeing.

#### 2.2.4 Future orientation

Five items administered at both timepoints examined how much the adolescents (i) think about how things might be in the future, (ii) can see their life 10 years from now, (iii) feel hopeless about their future, (iv) think their life is full of problems they cannot overcome, and (v) think their problems will dominate all of their life at both timepoints. Each item was rated on a continuous scale ranging from 0 = *Never true* to 100 = *Always true*. The first two items were taken from the “Future Outlook Inventory,” a tool developed and used in previous research on antisocial and criminal behavior in adolescents (Monahan et al., 2009; Petrich & Sullivan, 2020). The remaining three items, all reverse‐coded, were taken from the “Hope” subscale of the Intermediate Outcomes Measurement Instrument, a tool developed to assess young prisoners' future orientation (Maguire et al., 2019). CFA confirmed that the items loaded onto two factors, with the *χ* ^2^ test being nonsignificant (*χ* ^2^[4] = 5.19, *p* = .27, RMSEA = 0.07, *p* for RMSEA < .05 test = .34, SRMR = 0.03, CFI = 0.99, TLI = 0.98). Furthermore, the scale had excellent internal reliability (*ω* = 0.93, SE = 0.02, 95% CI: 0.90–0.96). Thus, the five items measuring future orientation were grouped into subscales of “future outlook” and “hope,” with the mean of the items comprising each subscale comprising that subscale's score. Higher scores in the future outlook and hope scales indicate a more positive future orientation.

#### 2.2.5 Positive experience

Five items examined how (i) happy, (ii) creative, (iii) excited, (iv) confident, and (v) part of a group, the adolescents felt while taking part in the dance sessions at both timepoints. At T1, the questions were asked with the instructions, “Imagining the upcoming dance sessions, how often would you feel…”. At T2, the questions were asked with the instructions “While at a dance session, how often did you feel…” Each item was rated on a 4‐point Likert scale (4 = *Always*, 3 = *Mostly*, 2 = *Rarel*y, 1 = *Never*). CFA confirmed that all five items loaded onto a single component, with the *χ* ^2^ test being nonsignificant (*χ* ^2^[5] = 7.27, *p* = .20, RMSEA = 0.09, *p* for RMSEA < .05 test = .27, SRMR = 0.07, CFI = 0.94, TLI = 0.88). Furthermore, the scale had good internal reliability (*ω* = 0.65, SE = 0.09, 95% CI: 0.49–0.82). Thus, the mean of all five items was taken to calculate the positive experience score, with higher scores indicating more positive experiences.

#### 2.2.6 Performance

Four items at T2 examined how satisfied the adolescents were with their own and their group's performance, and the degree to which their own and their group's performance matched their expectations (Cohen et al., 2023). Each item was rated on a continuous scale, with the satisfaction items' scale ranging from 0 = *Not at all* to 100 = *Very much*, and the expectation items' scale ranging from 0 = *Worse than expected* through 50 = *As expected* to 100 = *Better than expected*. For all items, higher scores indicated better perceived performance.

### 2.3 Statistical analyses

All statistical analyses were conducted in R Studio Version 2023.06.0+421 using the lavaan, lme4, mice, and tidyverse packages (Bates et al., 2015; van Buuren & Groothuis‐Oudshoorn, 2011; Wickham et al., 2019). Before conducting inferential analyses, scale reliability was assessed using T1 data from the original data set (as reported above). Omega values were calculated using the ci.reliability() function in R with 1000 bootstraps and standard bootstrap confidence intervals. CFA was conducted using the cfa() function. Predictor variables were scaled to SD = 1 and centered.

Hypotheses 1–3 were assessed with three linear mixed models, with group (intervention vs waitlist control) × time point (T1: baseline vs T2: post‐programme) as predictor variables, participant ID as the random effect variable, and wellbeing, social bonding, and future orientation as outcome variables, respectively. Hypotheses 4–6 were assessed using three linear regression models of the intervention group data. For Hypotheses 4 and 6, T2 – T1 difference score of the outcome variable was regressed on the T2 – T1 difference score of the predictor variable(s). For Hypothesis 5, T2 – T1 difference in social bonding was regressed on the T2 performance scores.

Given the high dropout rate from T1 to T2, multiple imputation was done using the mice package in R to improve the estimates obtained. The mice package produces multiple imputed data sets by replacing the missing values in the original data set using Gibbs sampling (van Buuren & Groothuis‐Oudshoorn, 2011). To replace the missing data points, T2 values are predicted by fitting a linear regression line based on T1 values. At each imputation, a random error is added such that the random errors of a given imputation have a mean = 0 and a standard deviation equal to that observed at T1. This method ensures that the imputed values are plausible and that the shape and spread of the original data and the population are preserved in the imputed data sets. For inferential analyses, we obtained 10 such imputed data sets, repeated the analyses in each of the 11 data sets (original data set + 10 imputed data sets), and pooled the model outputs to obtain a single result for each hypothesis. Pooling across model outputs was done by averaging the beta estimates, taking the square root of the sum of squares of standard errors, and calculating the corresponding *Z* scores and *p* values. Hypothesis tests are based on this highly powered pooled data set. A comparison of the results for each hypothesis across the data sets, including those of the original data set, is provided in Supporting Information S1: Figures S1 and S2. Data distributions of the original data set for each hypothesis are provided in Supporting Information S1: Figure S3. Hypothesis 5 analyses were not repeated in the imputed data sets as predictor variables for this hypothesis were collected at T2 only. The analysis scripts, including the multiple imputation calculations, can be found on the project's OSF page: https://osf.io/b5dfp/.

## 3 RESULTS

### 3.1 Group differences at T1

The intervention and waitlist control groups were similar in terms of gender, ethnicity, and age (see Table 1; all *p*'s > .05). Participants' T1 survey responses in the original data set were compared across the intervention and waitlist control groups with a simple linear regression. At baseline, participants in the intervention group did not differ significantly from participants in the waitlist control group in terms of social bonding (*M*_intv_ = 23.95, *M*_ctrl_ = 24.33, *β* = 0.38, SE = 5.24, *p* = .94), wellbeing (*M*_intv_ = 67.29, *M*_ctrl_ = 71.23, *β* = 3.94, SE = 4.27, *p* = .36), future outlook (*M*_intv_ = 68.03, *M*_ctrl_ = 63.09, *β* = −4.94, SE = 6.59, *p* = .46), or hope (*M*_intv_ = 68.25, *M*_ctrl_ = 74.67, *β* = 6.42, SE = 6.23, *p* = .31).

### 3.2 Randomness of attrition at T2

Comparison of T1 survey responses of participants who completed vs did not complete the T2 survey did not reveal significant differences in T1 social bonding (*M*_complete_ = 25.36, *M*_drop_ = 22.50, *β* = −2.86, SE = 5.27, *p* = .59), wellbeing (*M*_complete_ = 71.86, *M*_drop_ = 65.51, *β* = −6.36, SE = 4.25, *p* = .14), or future outlook (*M*_complete_ = 68.70, *M*_drop_ = 61.82, *β* = −6.88, SE = 6.61, *p* = .30). However, participants who completed the T2 survey scored significantly lower on hope at T1 as compared to participants who dropped out at T2 (*M*_complete_ = 76.98, *M*_drop_ = 63.65, *β* = −13.33, SE = 6.08, *p* = .03).

### 3.3 Hypothesis 1: Group dance and social bonding

**Figure 2:**
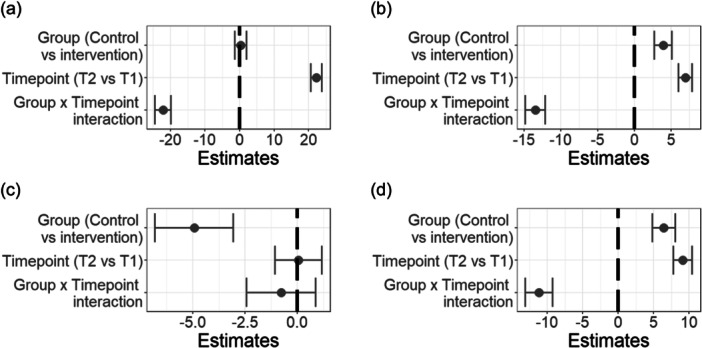
Model outputs of the pooled results of the imputed data sets for Hypotheses 1–3 examining the interaction of timepoint (T1 vs T2) and group (intervention vs waitlist control). Outcome variables are: (a) social bonding, (b) wellbeing, (c) future outlook, and (d) hope. Positive estimates indicate a positive association between the predictors (labels on the *y*‐axis) and the outcome variable (labels on individual figure headings), negative estimates indicate a negative association and the error bars indicate the standard error of the estimate.

There was no main effect of group (*β* = .38, SE = 1.69, *p* = .82), but a main effect of timepoint such that participants reported higher bonding at T2 as compared to at T1 (*β* = 22.26, SE = 1.57, *p* < .0001; Figure 2a). Supporting Hypothesis 1, the interaction of group × timepoint was significant (*β* = −22.14, SE = 2.30, *p* < .0001), indicating a greater increase in social bonding from T1 to T2 in the intervention vs control group (*β* = 22.79, SE = 1.78, *p* < .0001; Figure 1 = 0.75; Figure 2a).

### 3.4 Hypothesis 2: Group dance and wellbeing

There were main effects of group and timepoint, such that participants in the intervention group had less positive wellbeing than participants in the waitlist control group (*β* = 3.94, SE = 1.21, *p* = .001) and wellbeing was higher in T2 than in T1 (*β* = 6.97, SE = 0.93, *p* < .0001). Supporting Hypothesis 2, the interaction of group × timepoint was significant (*β* = −13.49, SE = 1.36, *p* < .0001; Figure 2b), indicating that wellbeing increased from T1 to T2 in the intervention group (*β* = 8.28, SE = 1.16, *p* < .0001; Figure 2b), while a decrease in wellbeing (*β* = −6.63, SE = 1.43, *p* < .0001) was observed in the waitlist control group. These results—that is, the main effects of group and timepoint and group × timepoint interaction—were repeated across each of the five subscales of wellbeing, namely Positive emotions, Engagement, Relationships, Meaning and Accomplishment (see Supporting Information S1: Table S2).

### 3.5 Hypothesis 3: Group dance and future orientation

For the future outlook dimension of future orientation, there was a main effect of group (*β* = −4.94, SE = 1.88, *p* = .008), indicating that across timepoints, participants in the intervention group had a more positive future outlook as compared to participants in the waitlist control group. There was no significant main effect of timepoint (*β* = .05, SE = 1.12, *p* = .96), and, contrary to Hypothesis 3, no group × timepoint interaction (*β* = −.78, SE = 1.65, *p* = .64; Figure 2c) was found.

For the hope dimension of future orientation, there were main effects of group and timepoint such that, across timepoints, participants in the intervention group had less hope about the future than participants in the waitlist control group (*β* = 6.42, SE = 1.63, *p* < .0001), and that, across groups, hope increased from T1 to T2 (*β* = 9.11, SE = 1.31, *p* < .0001). Supporting Hypothesis 3, these main effects were qualified by a significant group × timepoint interaction (*β* = −11.15, SE = 1.93, *p* < .0001; see Figure 2d), indicating that hope increased from T1 to T2 in the intervention group, (*β* = 8.90, SE = 1.61, *p* < .0001), while it decreased from T1 to T2 in the waitlist control group (*β* = −2.81, SE = 1.60, *p* = .08).

### 3.6 Hypothesis 4: Social bonding, positive experience, and wellbeing

**Figure 3:**
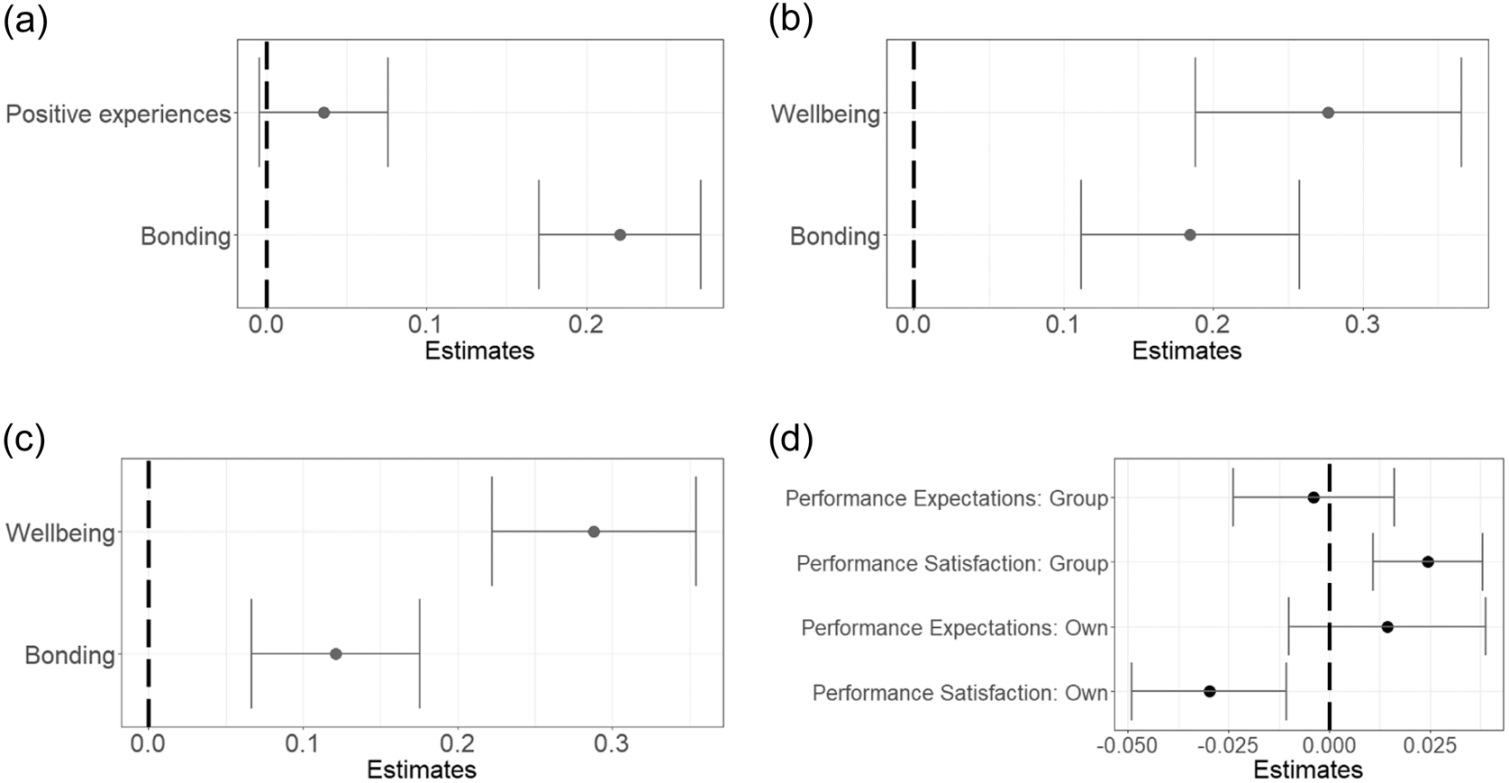
Model outputs of the pooled results of the imputed data sets for Hypotheses 4 and 5 and original data set results for Hypothesis 6 examining changes in wellbeing, future orientation, social bonding, and performance within the intervention group. Outcome variables are T2 – T1 difference in: (a) wellbeing, (b) future outlook, (c) hope, and (d) social bonding. Positive (negative) estimates indicate a positive (negative) association between the predictors on the *y*‐axis and the outcome variable.

There was no significant association of positive experiences (*β* = .04, SE = 0.04, *p* = .38; Figure 3a), but a significant positive association of bonding with wellbeing (*β* = .22, SE = 0.05, *p* = .00001; Figure 3a).

### 3.7 Hypothesis 5: Wellbeing, social bonding, and future orientation

Supporting Hypothesis 5, increases in wellbeing and bonding were associated with improved future outlook (Wellbeing: *β* = .28, SE = 0.09, *p* = .002, Bonding: *β* = .18, SE = 0.07, *p* = .01; Figure 3b), and increased hope (Wellbeing: *β* = −.30, SE = 0.07, *p* = .0002, Bonding: *β* = −.12, SE = 0.06, *p* = .03; Figure 3c).

### 3.8 Hypothesis 6: Group performance and social bonding

Contrary to Hypothesis 6, none of the performance variables significantly predicted social bonding differences (group performance expectations: *β* = −.03, SE = 0.02, *p* = .14, Group performance satisfaction: *β* = .02, SE = 0.01, *p* = .10, Own performance expectations: *β* = .01, SE = 0.02, *p* = .57, Own performance satisfaction: *β* = −.004, SE = 0.02, *p* = .84; Figure 3d).

## 4 DISCUSSION

This study examined longitudinal links among adolescents' social bonding, wellbeing, and future outlook during an online dance program. Supporting our theoretical model, engaging in a 5‐week group dance program improved the adolescents' social bonding, wellbeing, and future orientation significantly more as compared to being on a waiting list. Improvements in program participants' wellbeing were associated with increases in self‐reported bonding with other participants at the end of the program. Analyses also revealed that increases in wellbeing and social bonding were associated with a more positive future outlook.

These findings support the suggestion that group dance can benefit social and mental health outcomes (Fancourt et al., 2021). Combining measures of social bonding, wellbeing, and future orientation in a longitudinal, randomized controlled intervention design and analyzing hypothesized relationships among them, this study goes beyond simply describing outcomes to investigating how these outcomes are interlinked. In prior research, several suggestions have been made about how group dance incurs benefits ranging from increasing creativity and boosting self‐confidence to encouraging physical activity and facilitating positive emotions (Tao et al., 2022; Zarobe & Bungay, 2017). Our study adds an important social dimension to this literature, indicating that the social bonds created during group dance are associated with an increase in wellbeing. The results expand previous research on the critical role of social bonds in supporting mental health and wellbeing (Solar & Irwin, 2010; Viner et al., 2012).

Performance‐based social activities, such as dance, can be associated with self‐imposed or socially‐imposed pressures to achieve a certain standard in front of others, and a sense of not “measuring up” might impact negatively on social connection with the group. However, contrary to our predictions, in our study, the participants' own or group performance was not significantly associated with social bonding. This might be because the participants engaged with the program online, and they were not working towards an end‐of‐program performance. The online medium may have reduced the participants' ability to gauge performance, and, consequently, our ability to detect an association between doing *better* than expected as a group and the creation of stronger group bonds. In addition, the weekly use of breakout rooms, where participants shared something about their day and checked in with each other, may have enhanced social closeness. The lack of an end‐of‐program performance may have diverted the participants' focus away from performance‐related measures. It is important to note that the performance analyses were conducted only in the original data set (i.e., not in the imputed data sets), and they are, therefore, likely to be underpowered and need further corroboration in future research.

If there is no association between perceptions of performance and social bonding, then this could be interesting for providers and stakeholders in relation to, for example, recruitment and program design. It would suggest that sharing an overarching, interdependent goal, such as an end‐of‐program performance or competition, as in sports teams, which helps boost identity building among group members (Cohen et al., 2023; Jetten et al., 2014), is not necessary for social bonding and wellbeing effects to occur. Simply gathering with others to move together might be sufficient to induce the observed positive effects. This is in line with a growing literature on behavioral synchrony that has revealed that people who merely move their bodies in time to the same rhythm with others feel more affiliated, trusting, and cooperative towards each other (Rennung & Göritz, 2016). However, it may be harder to perceive the synchrony between dancers via an online medium; hence, it will be important for future research to examine how perceived and actual synchrony between dancers relate to social bonding in online and in‐person delivery. Another mechanism mentioned in the literature, which our data do not support, is the role of positive emotions and experiences in wellbeing (Tao et al., 2022; Zarobe & Bungay, 2017). Anecdotally, however, one of the participants who joined our program each week from a mental health hospital with her occupational nurse reported gaining the confidence to join an in‐person activity group when she came out of the hospital because of her positive experience with this program. Thus, it is possible that our measures could not capture aspects of personal development that occurred, a point future research should explore further.

Our data collection took place about one year into the Covid‐19 pandemic and during a lockdown period. The Covid‐19 pandemic and subsequent economic struggles have brought societal uncertainties, physical health concerns, and disruptions to education and daily life, which led to increased feelings of loneliness, poor mental wellbeing, and reduced future orientation in the adolescent population across the globe (Carey et al., 2023; Saulle et al., 2022; Skinner et al., 2022; Zolopa et al., 2022). This widescale data suggest that (i) mental disorders tend to occur when individuals experience a lack of social bonds and (ii) fostering social bonds can help sustain wellbeing (Tunçgenç, van Mulukom & Newson, 2023). Key to this is enrolling widely applicable preventive interventions before children reach their teenage years. Following the social prescribing approach, which advocates the use of social groups as a resource for better health (Drinkwater et al., 2019; Jetten et al., 2012), a longitudinal study on the Arts on Prescription program delivered to adolescents aged 13–16 years in England was shown to improve wellbeing and resilience (Efstathopoulou & Bungay, 2021). Such low‐intensity interventions, which do not rely on specialists, and are typically delivered in school or community settings, offer promise for widescale applicability. Extending these findings, our study reveals the promise of online dance programs, which would open new avenues for improving scalability and accessibility (Ateca‐Amestoy & Castiglione, 2023). By revealing the social bonding pathway through which group dance can improve wellbeing in an online intervention, this study addresses the limitations of existing social prescribing research (Blodgett et al., 2022; Hsueh et al., 2022; Vidovic et al., 2021) and supports future applications of group dance interventions in adolescents.

Although online mediums are relatively new and rarely used as interventions to improve wellbeing, systematic reviews conducted both before and after the Covid‐19 pandemic have shown that they offer promise (Clarke et al., 2015; Gkatsa, 2023; Moss et al., 2023). In particular, it was found that online physical activity interventions improved physical health and muscle strength, art‐based online interventions alleviated inattention, and online skills‐based interventions enhanced adolescents' mental health. Increased likelihood of the participants dropping out of online interventions was reported as a key drawback (Clarke et al., 2015), which our study was not immune to either. Consequently, our relatively small sample size, comprised mostly of girls, limits generalizability and calls for future replications of our findings.

It is also possible that the program and data collection taking place online may have impacted participants' adherence and fidelity of delivery (Sanetti & Kratochwill, 2009). To ensure data quality was not impacted, the first author (B. T.) was always present in the data collection sessions. Nevertheless, we had no control over the activities that the waitlist control group may have been involved with while they waited for their program to commence, which is important for future research to consider. To increase adherence and fidelity, and to further delineate the unique benefits incurred from different components of the dance program (e.g., the degree of synchronization between dancers), in‐person studies might be beneficial.

A strength of this research is that it was co‐produced with the dance company that delivered the program to the adolescents. This entailed identifying the research priorities, selecting the measurement tool, and designing and writing up the study together. Involving the stakeholders in the study process increases the chances of the research findings translating into real‐life impact (Arnstein, 1969). Our findings provide proof‐of‐concept for the social bonding and wellbeing benefits of an online dance program and point out future considerations for improvement (e.g., regarding participant dropout and adherence and delivery fidelity).

## 5 CONCLUSION

In conclusion, we show that a brief, 5‐week‐long group dance program helped adolescents form stronger social connections, which was associated with improved wellbeing and future orientation. This study provides evidence that online dance activities can work even at times of mass social isolation, which opens up new possibilities for social prescribing. Forging social bonds through group dance activities could be a vital and cost‐effective route to improving adolescent wellbeing and buffering the detrimental effects of loneliness.

## Supporting information

Supporting information.

## Data Availability

The data that support the findings of this study are openly available in the Open Science Framework at https://osf.io/b5dfp/.
