# Supplementary material for: Benefits of an online group dance program for adolescents' social bonding and wellbeing
Source: J Adolesc. 2024 Aug 15;96(8):1917–28. doi: 10.1002/jad.12391 (PMC11618708; doi:10.1002/jad.12391)
Supplement: Supplementary file 1 — Supporting information. [file JAD-96-1917-s001.docx]

**Benefits of an online group dance program for adolescents’ social bonding and wellbeing**

**Supplementary Results**

1. **Descriptive statistics of the imputed datasets**

| *Table S1. Descriptive statistics (M, SD) of key variables from the imputed datasets.* | | | |
| --- | --- | --- | --- |
|  | **T2 Intervention** | **T2 Waitlist** |  |
| **Imputed Dataset 1** |  |  |  |
| **Social Bonding** | 48.21 (26.61) | 29.48 (25.86) |  |
| **Wellbeing** | 74.74 (10.93) | 63.35 (15.85) |  |
| **Future Outlook** | 69.71 (19.85) | 68.15 (21.95) |  |
| **Hope** | 75.89 (17.23) | 30.33 (16.32) |  |
| **Imputed Dataset 2** |  |  |  |
| **Social Bonding** | 43.78 (25.23) | 25.12 (20.84) |  |
| **Wellbeing** | 74.64 (11.44) | 63.85 (16.72) |  |
| **Future Outlook** | 68.02 (22.19) | 65.69 (22.29) |  |
| **Hope** | 74.39 (17.36) | 71.15 (15.50) |  |
| **Imputed Dataset 3** |  |  |  |
| **Social Bonding** | 45.90 (22.02) | 29.42 (21.35) |  |
| **Wellbeing** | 74.53 (10.80) | 65.70 (16.43) |  |
| **Future Outlook** | 71.10 (21.75) | 65.35 (20.76) |  |
| **Hope** | 74.34 (17.49) | 71.40 (14.64) |  |
| **Imputed Dataset 4** |  |  |  |
| **Social Bonding** | 43.77 (25.19) | 22.16 (18.53) |  |
| **Wellbeing** | 72.77 (12.17) | 63.84 (15.45) |  |
| **Future Outlook** | 66.60 (19.38) | 62.70 (20.98) |  |
| **Hope** | 76.00 (15.91) | 69.60 (14.99) |  |
| **Imputed Dataset 5** |  |  |  |
| **Social Bonding** | 49.34(24.87) | 22.24 (18.75) |  |
| **Wellbeing** | 72.30 (12.50) | 65.90 (17.34) |  |
| **Future Outlook** | 62.35 (20.61) | 58.67 (22.28) |  |
| **Hope** | 79.63 (18.62) | 71.52 (15.23) |  |
| **Imputed Dataset 6** |  |  |  |
| **Social Bonding** | 42.72 (22.73) | 24.34 (19.43) |  |
| **Wellbeing** | 75.16 (10.39) | 65.54 (15.91) |  |
| **Future Outlook** | 67.21 (24.14) | 62.57 (23.80) |  |
| **Hope** | 74.83 (15.50) | 72.64 (15.37) |  |
| **Imputed Dataset 7** |  |  |  |
| **Social Bonding** | 44.09 (24.49) | 23.27 (16.52) |  |
| **Wellbeing** | 73.11 (11.81) | 63.73 (15.86) |  |
| **Future Outlook** | 68.27 (21.68) | 63.44 (21.63) |  |
| **Hope** | 75.73 (18.17) | 72.60 (15.82) |  |
| **Imputed Dataset 8** |  |  |  |
| **Social Bonding** | 45.53 (24.90) | 20.98 (19.17) |  |
| **Wellbeing** | 73.89 (11.57) | 64.19 (15.80) |  |
| **Future Outlook** | 71.39 (21.15) | 59.89 (23.03) |  |
| **Hope** | 77.84 (18.00) | 70.17 (17.03) |  |
| **Imputed Dataset 9** |  |  |  |
| **Social Bonding** | 48.40 (24.38) | 28.51 (23.08) |  |
| **Wellbeing** | 73.98 (11.47) | 64.03 (15.80) |  |
| **Future Outlook** | 66.97 (22.69) | 59.81 (21.82) |  |
| **Hope** | 76.24 (16.89) | 68.77 (17.30) |  |
| **Imputed Dataset 10** |  |  |  |
| **Social Bonding** | 44.44 (22.92) | 27.56 (22.42) |  |
| **Wellbeing** | 74.17 (11.19) | 66.72 (16.59) |  |
| **Future Outlook** | 69.53 (21.85) | 59.61 (25.40) |  |
| **Hope** | 77.92 (16.70) | 77.44 (17.02) |  |

1. **Comparison of the results across the original and imputed datasets**

| 1. Social Bonding   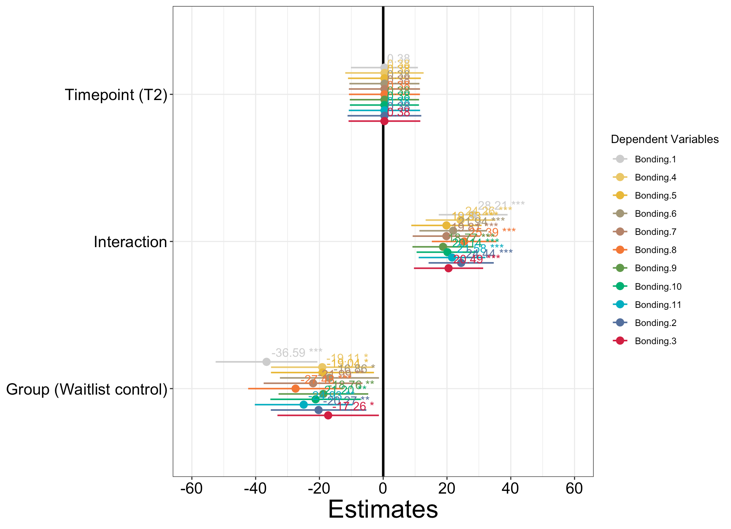 | 1. Wellbeing   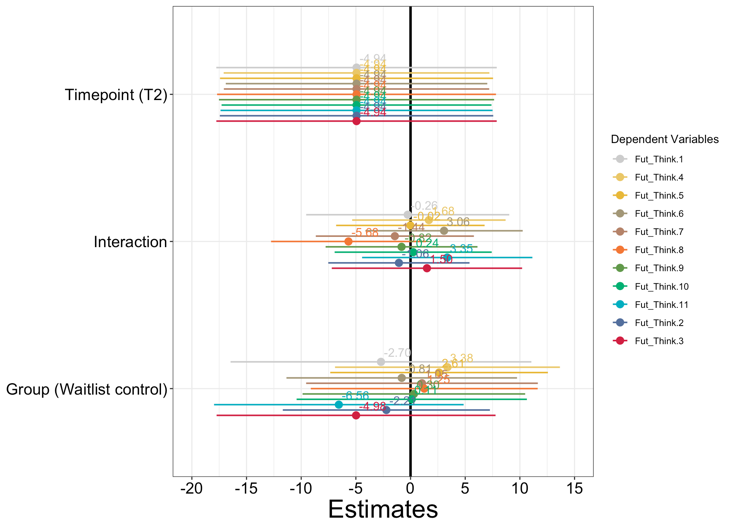 |
| --- | --- |
| 1. Future Outlook   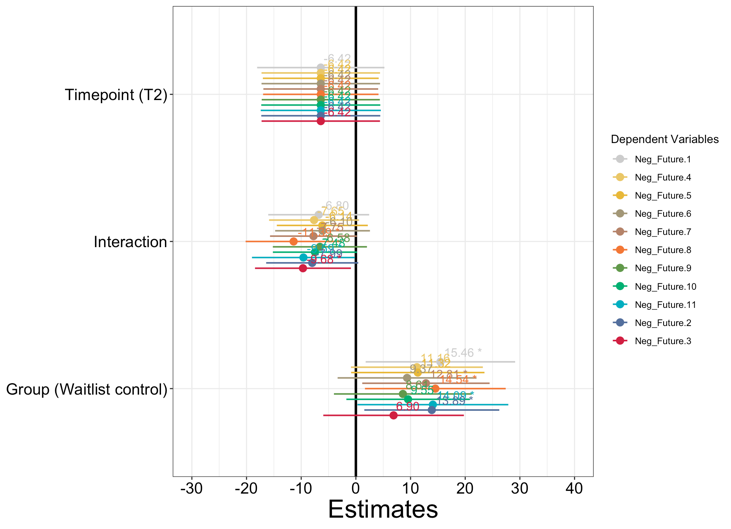 | 1. Hope   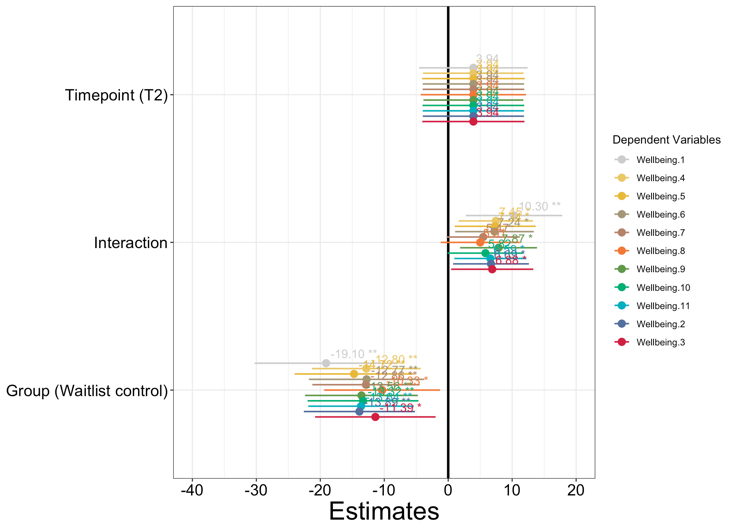 |
| ***Figure S1. Model outputs of each of the imputed datasets for outcome variables (A) social bonding, (B) wellbeing (C) future outlook, and (D) hope.*** Dots indicate point estimates, bars indicate standard error, and each colour indicates a different imputed dataset. | |

1. **Replication of all findings within the original dataset**

Figure S2 shows box plots of depicting how variables changed over time as hypothesised in Hypothesis 1 (Figure S2A), Hypothesis 2 (Figure S2B) and Hypothesis 3 (Figure S2C-D) within the original dataset.

Figure S3 shows associations between variables changing over time as hypothesised in Hypothesis 4 (Figure S3A), Hypothesis 5 (Figure S3B-C) and Hypothesis 6 (Figure S3D) within the original dataset.

| (A)  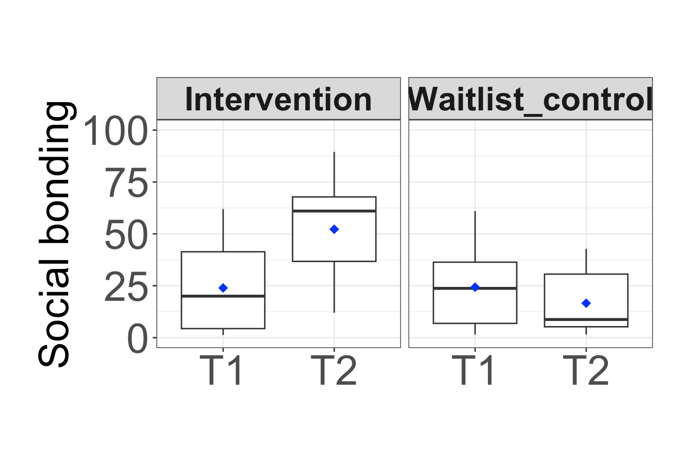 | (B)  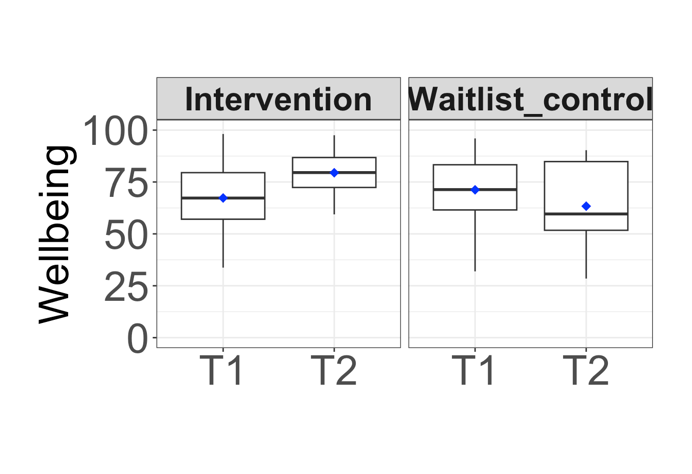 |
| --- | --- |
| (C)  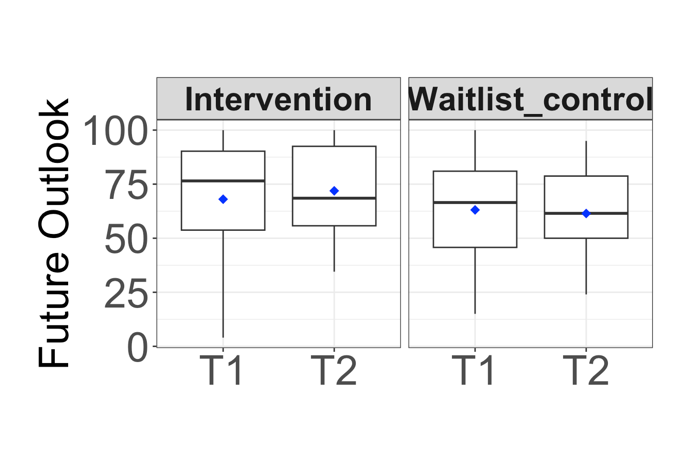 | (D)  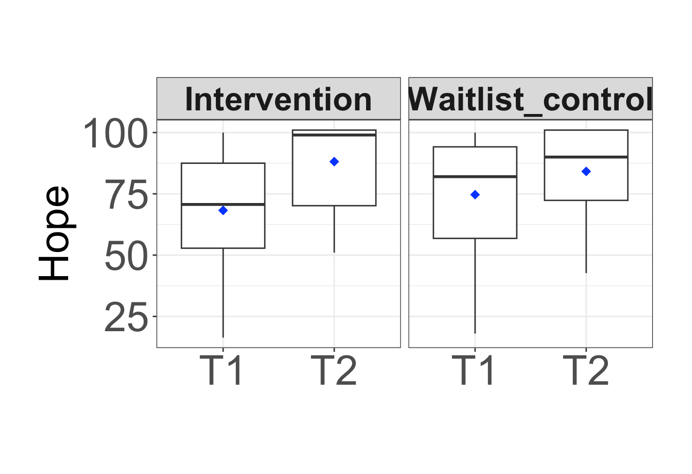 |
| ***Figure S2. Box plots showing data distributions within the original dataset for (A) social bonding, (B) wellbeing, (C) future outlook, and (D) hope.*** In all figures, blue dots indicate the mean, bars indicate standard error, and horizontal lines indicate the median. | |

| (A)  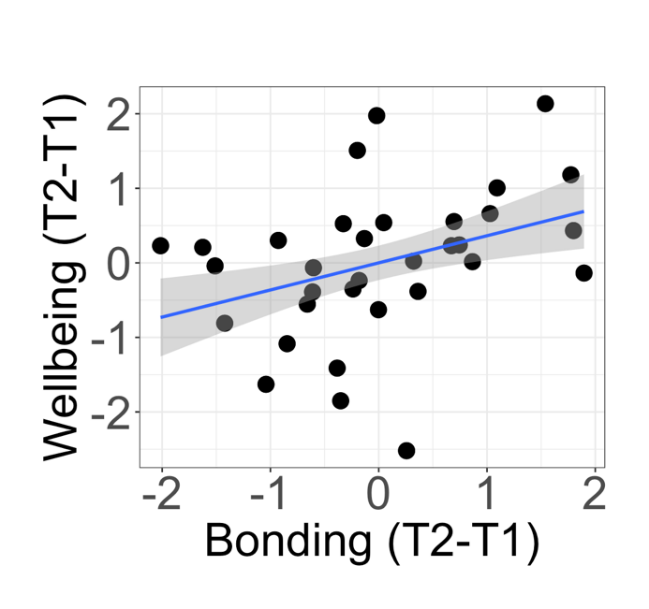 | (B)  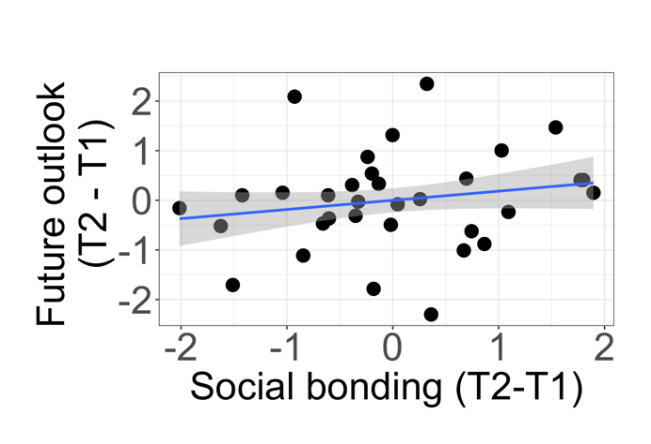  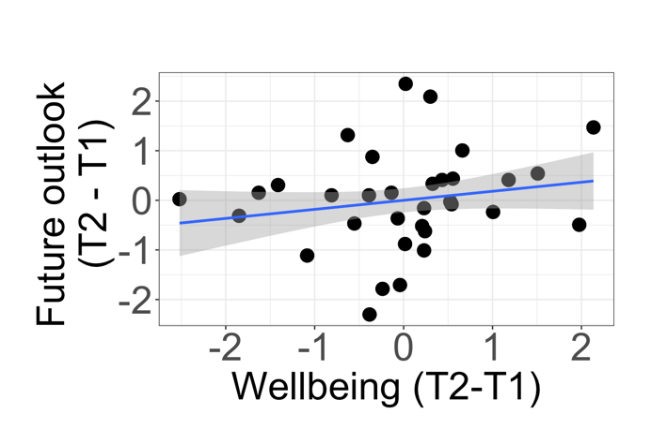 |
| --- | --- |
| (C)  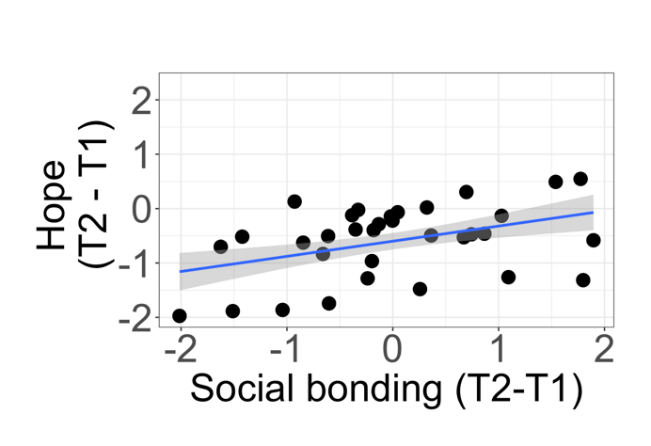  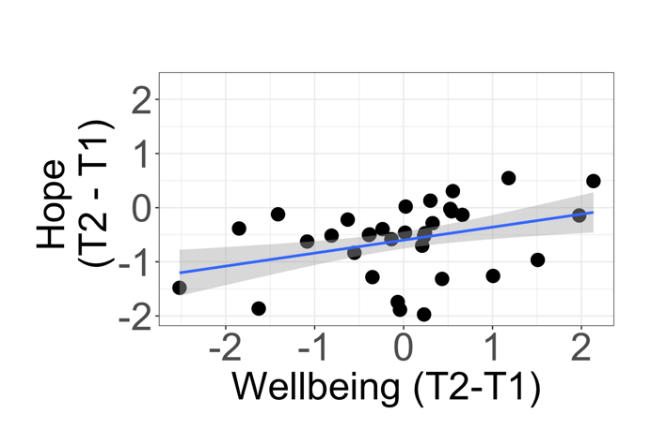 | (D)  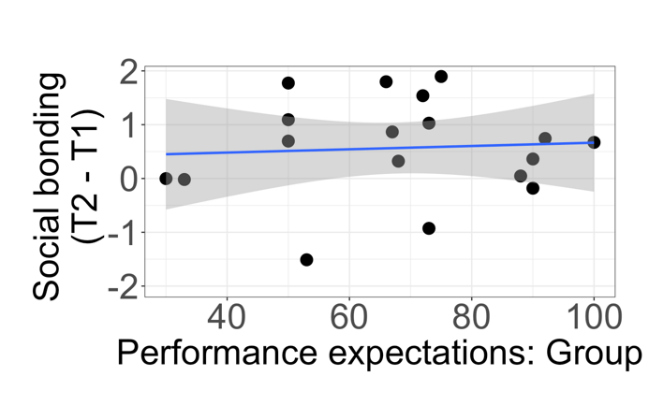  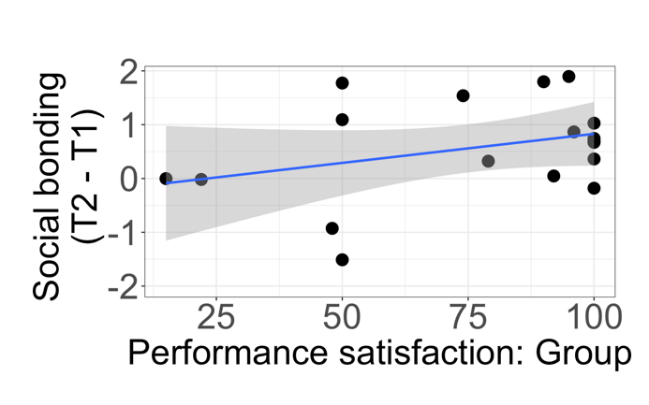 |
| ***Figure S3. Scatter plots showing hypothesised bivariate change over time (T2 – T1) associations within the original dataset between (A) social bonding and wellbeing, (B) bonding, wellbeing and future outlook, (C) bonding, wellbeing and hope, and (D) group performance variables and social bonding.*** In all figures, black dots indicate each data point, blue line indicates best linear fit, and grey shades indicate 95% confidence intervals. | |

**Hypothesis 1: Group Dance & Social Bonding**. Linear mixed models regressing bonding on group (intervention vs waitlist control) x timepoint (T1 vs T2) revealed no main effect of group (β = 0.38, *SE* = 5.24, *p* = 0.94), but main effect of timepoint such that children reported higher bonding at T2 as compared to at T1 (β = 28.21, *SE* = 5.29, *p* < 0.0001). In addition, the interaction of group x timepoint was significant (β = -36.59, *SE* = 7.82, *p* = 0.0001), indicating that bonding increased from T1 to T2 in the intervention group (β = 28.27, *SE* = 6.52, *p* < 0.0001), while no significant change was observed in the waitlist control group (β = -7.73, *SE* = 5.52, *p* = 0.17).

**Hypothesis 2: Group Dance & Wellbeing**. Linear mixed models regressing wellbeing on group (intervention vs waitlist control) x timepoint (T1 vs T2) revealed main effects of group and timepoint such that children in the intervention group had significantly higher wellbeing as compared to children in the waitlist control group (β = 23.04, *SE* = 8.19, *p* = 0.007), and children had higher wellbeing at T2 as compared to at T1 (β = 10.30, *SE* = 3.70, *p* = 0.009). In addition, the interaction of group x timepoint was significant (β = -19.10, *SE* = 5.48, *p* = 0.002), indicating that wellbeing increased from T1 to T2 in the intervention group (β = 12.17, *SE* = 4.39, *p* = 0.008), while no significant change (β = -7.88, *SE* = 5.66, *p* = 0.17) was observed in the waitlist control group. Note that the latter finding is contrary to the pooled results.

**Hypothesis 3: Group Dance & Future Orientation**. Linear mixed models regressing the first dimension of future orientation (i.e., future outlook) on group (intervention vs waitlist control) x timepoint (T1 vs T2) revealed no significant effects of group (β = -2.24, *SE* = 10.69, *p* = 0.84), timepoint (β = -0.26, *SE* = 4.55, *p* = 0.96,) or group x timepoint interaction (β = -2.70, *SE* = 6.74, *p* = 0.69) on the future outlook variable. Note that the former finding is contrary to the pooled results.

A linear mixed models regressed on the second dimension of future orientation (i.e., hope) revealed main effects of group and timepoint such that, across timepoints, children in the intervention group did not differ from children in the waitlist control group (β = 3.94, *SE* = 4.24, *p* = 0.36), but that, across groups, hope increased from T1 to T2 (β = 10.30, *SE* = 3.70, *p* = 0.01). These main effects were qualified by a significant group x timepoint interaction (β = -19.10, *SE* = 5.48, *p* = .002), indicating that for children in the intervention group, hope increased from T1 to T2 (β = 12.17, *SE* = 4.39, *p* = 0.008), while no significant change (β = -7.88, *SE* = 5.66, *p* = 0.17) was observed in the waitlist control group. Note that the latter finding is contrary to the pooled results.

**Hypothesis 4: Social Bonding, Positive Experience & Wellbeing**. A linear model with the outcome variable being the T2 – T1 difference score in wellbeing, and the predictor variables being the T2 – T1 difference scores in social bonding and positive experience was conducted. No significant effects of bonding (β = 0.36, *SE* = 0.24, *p* = 0.15) or of positive experience were found on wellbeing (β = -0.13, *SE* = 0.17, *p* = 0.44). Note that the former finding is contrary to the pooled results.

**Hypothesis 5: Wellbeing, Social Bonding & Future Orientation**. Two linear models were conducted, one for each dimension of the future orientation variable (i.e., future outlook and hope), to examine how pre-post differences in wellbeing and social bonding would predict pre-post differences in future orientation. The results revealed no association of increased wellbeing and bonding with improved future outlook or hope (**future outlook**: Wellbeing: β = 0.36, *SE* = 0.46, *p* = 0.45, Social Bonding: β = 0.17, *SE* = 0.37, *p* = 0.65; **hope**: Wellbeing: β = -0.40, *SE* = 0.32, *p* = 0.23, Social Bonding: β = -0.12, *SE* = 0.26, *p* = 0.65). Note that these findings are contrary to the pooled results.

1. **Replication of Hypothesis 2 findings within the PERMA wellbeing sub-scales**

| *Table S2. Pooled results of N = 11 samples examining the effects of Group (intervention vs waitlist control) x Timepoint (T1 vs T2) on the 5 dimensions of the PERMA wellbeing scale.* | | | | | | |
| --- | --- | --- | --- | --- | --- | --- |
|  | **Entire Sample** | | | **Intervention** | | **Control** |
|  | **Group** | **Timepoint** | **Group * Timepoint** | **Timepoint** | **Timepoint** | |
| **Positive Emotions** | β = 2.10  *SE* = 1.53  *p* < .0001 | β = 3.17  *SE* = 1.19  *p* = 0.008 | β = -10.69  *SE* = 1.75  *p* < .0001 | β = 5.18  *SE* = 1.48  *p* = .0005 | β = -7.38  *SE* = 1.78  *p* < .0001 | |
| **Engagement** | β = 7.71  *SE* = 1.25  *p* < .0001 | β = 9.77  *SE* = 1.05  *p* < .0001 | β = -15.84  *SE* = 1.55  *p* < .0001 | β = 10.90  *SE* = 1.24  *p* < .0001 | β = -6.60  *SE* = 1.37  *p* < .0001 | |
| **Relationships** | β = -1.99  *SE* = 1.42  *p* = 0.16 | β = 2.84  *SE* = 1.18  *p* = 0.02 | β = -8.45  *SE* = 1.73  *p* < .0001 | β = 4.18  *SE* = 1.37  *p* = .002 | β = -5.56  *SE* = 1.62  *p* = .0006 | |
| **Meaning** | β = 5.56  *SE* = 1.64  *p* < .0001 | β = 10.30  *SE* = 1.33  *p* < .0001 | β = -17.77  *SE* = 1.96  *p* < .0001 | β = 11.43  *SE* = 1.57  *p* < .0001 | β = -8.15  *SE* = 1.93  *p* < .0001 | |
| **Accomplishment** | β = 6.31  *SE* = 1.42  *p* < .0001 | β = 8.87  *SE* = 1.13  *p* < .0001 | β = -14.73  *SE* = 1.66  *p* < .0001 | β = 9.69  *SE* = 1.40  *p* < .0001 | β = -5.44  *SE* = 1.61  *p* = .0007 | |
